# Myelin Abnormalities in the Optic and Sciatic Nerves in Mice With GM1-Gangliosidosis

**DOI:** 10.1177/1759091415568913

**Published:** 2015-02-13

**Authors:** Karie A. Heinecke, Adrienne Luoma, Alessandra d’Azzo, Daniel A. Kirschner, Thomas N. Seyfried

**Affiliations:** 1 Department of Biology, Boston College, Chestnut Hill, MA, USA; 2 Department of Biochemistry and Molecular Biology, Committee on Immunology, University of Chicago, IL, USA; 3 Department of Genetics, St. Jude Children’s Research Hospital, Memphis, TN, USA

**Keywords:** cerebrosides, gangliosides, lipids, X-ray diffraction

## Abstract

GM1-gangliosidosis is a glycosphingolipid lysosomal storage disease involving accumulation of GM1 and its asialo form (GA1) primarily in the brain. Thin-layer chromatography and X-ray diffraction were used to analyze the lipid content/composition and the myelin structure of the optic and sciatic nerves from 7- and 10-month old β-galactosidase (*β-gal*) +/? and *β-gal* −/− mice, a model of GM1gangliosidosis. Optic nerve weight was lower in the *β-gal* −/− mice than in unaffected *β-gal* +/? mice, but no difference was seen in sciatic nerve weight. The levels of GM1 and GA1 were significantly increased in both the optic nerve and sciatic nerve of the *β-gal* −/− mice. The content of myelin-enriched cerebrosides, sulfatides, and plasmalogen ethanolamines was significantly lower in optic nerve of *β-gal* −/− mice than in *β-gal* +/? mice; however, cholesteryl esters were enriched in the *β-gal* −/− mice. No major abnormalities in these lipids were detected in the sciatic nerve of the *β-gal* −/− mice. The abnormalities in GM1 and myelin lipids in optic nerve of *β-gal* −/− mice correlated with a reduction in the relative amount of myelin and periodicity in fresh nerve. By contrast, the relative amount of myelin and periodicity in the sciatic nerves from control and *β-gal* −/− mice were indistinguishable, suggesting minimal pathological involvement in sciatic nerve. Our results indicate that the greater neurochemical pathology observed in the optic nerve than in the sciatic nerve of *β-gal* −/− mice is likely due to the greater glycolipid storage in optic nerve.

## Introduction

Lysosomal storage diseases (LSD) are characterized by the accumulation of macromolecules in the lysosomal compartment due to defects in catabolic enzymes. GM1-gangliosidosis is a type of LSD, caused by an autosomal recessive deficiency of lysosomal acid β-galactosidase (*β-gal* or *GLB1*), leading to accumulation of GM1 ganglioside and to a lesser extent of its asialo derivative GA1 in the brain and other tissues, followed by progressive neurodegeneration (O’Brien et al., 1965; Suzuki et al., 1968; Hahn et al., 1997; Suzuki et al., 2001; National Institute of Neurological Disorders and Stroke [NINDS], 2011). The most severe form of this disease (infantile or type I) has an early onset and is characterized by rapid neurological deterioration with death usually occurring before 3 years of age in humans (NINDS, 2011). There is currently no effective treatment for GM1-gangliosidosis. Besides humans, the disease can be found in other animals, including dogs, cats, and American black bear (Suzuki et al., 1968; Baker and Lindsey, 1974; Read et al., 1976; Muthupalani et al., 2014). The available knockout mouse models replicate many features of GM1-gangliosidisis in humans, including biochemical deficiency, neurochemical accumulation, and the ensuing loss of gross motor skills, visual impairment, and alterations in brain lipids (Hahn et al., 1997; Matsuda et al., 1997a, 1997b; Suzuki et al., 2001; Tessitore et al., 2004; Denny et al., 2007; Sano et al., 2009; Baek et al., 2010).

Lipids can provide important information about the integrity of the brain and nervous tissue. Gangliosides are sialic acid-containing glycosphingolipids residing in cell membranes, primarily in the nervous system (Ledeen, 1983). The individual ganglioside species are differentially distributed in the different cell types of the nervous system (Seyfried and Yu, 1984; Wiegandt, 1995; Vajn et al., 2013). GM1 ganglioside, cerebrosides, and sulfatides are enriched in myelin membranes (Menkes et al., 1966; Holm and Mansson, 1974; Yu and Yen, 1975; Zalc et al., 1981; Coetzee et al., 1996; Muse et al., 2001; Vajn et al., 2013). Cerebrosides, sulfatides, and plasmalogen ethanolamines are essential for proper myelination of axons (Farooqui and Horrocks, 2001; Marcus et al., 2006; Jackman et al., 2009; Chrast et al., 2011; Hayashi et al., 2013). Gangliosides GT1b and GD1a are involved in axonal-myelin stability (Vyas et al., 2002; Jackman et al., 2009), and ganglioside GQ1b plays a role in synaptic plasticity and calcium regulation of the myelin membranes (Sucher et al., 1991; Micu et al., 2006; Shin et al., 2014). Previous studies have shown major alterations to brain gangliosides, cerebrosides, and sulfatides in *β-gal*−/− mice, which simulate the abnormalities in humans with GM1-gangliosidosis (O’Brien and Sampson, 1965; Hahn et al., 1997; Matsuda et al., 1997b; Kasperzyk et al., 2004; Tessitore et al., 2004; Kasperzyk et al., 2005; Broekman et al., 2007; Denny et al., 2007; Baek et al., 2010).

Primary myelination is completed early in development; however, the amount of myelin in humans and rodents continues to increase with age in the central nervous system (CNS) (Menkes et al., 1966; Horrocks, 1973; Yu and Yen, 1975; Chrast et al., 2011). The lipid content in optic (CNS) and sciatic (peripheral nervous system [PNS]) nerves is similar to other tissues of the nervous system, in that they contain the major gangliosides, phospholipids, cholesterol, cerebrosides, and sulfatides (Peress and Boyle, 1975; Larrouquere-Regnier et al., 1979; Ganser et al., 1988a, 1988b; McNally et al., 2007; Acar et al., 2012). GM1 ganglioside is enriched in myelin and has been used as an indicator of myelin content in brain tissue (MacBrinn and O’Brien, 1969; Seyfried and Yu, 1980; Seyfried et al., 1984; Seyfried and Yu, 1984; Muse et al., 2001; Suzuki et al., 2001). The brains of animals and humans with GM1-gangliosidosis have shown defects in myelination (Kasama and Taketomi, 1986; Kaye et al., 1992; Folkerth et al., 2000; van der Voorn et al., 2005; Nada et al., 2011).

X-ray diffraction (XRD) has been used to determine myelin periodicity, relative amount of myelin, and membrane packing in freshly dissected nerves (Avila et al., 2005; Yin et al., 2006; Kirschner et al., 2010). Myelin periodicity refers to the width of the pair of membranes that constitutes the repeating structural unit in the multilamellar sheath. Membrane packing refers to interactions between the individual apposing surfaces (extracellular or intracellular) of the myelin membranes. XRD has been useful in identifying myelin membrane packing abnormalities in the nerves of animals with myelinating disorders (Kirschner and Sidman, 1976; Chia et al., 1984; Inouye et al., 1985; Mateu et al., 1991; Karthigasan et al., 1996; Vonasek et al., 2001; Avila et al., 2005; Yin et al., 2006; Avila et al., 2010). McNally et al*.* (2007) reported that the amount of myelin was reduced in optic nerves but not in sciatic nerves of SD mice. While many LSD display PNS involvement, McNally et al*.* were the first to analyze myelin in Sandhoff disease using XRD. Histological and imaging studies on GM1-gangliosidosis in humans suggest various neuropathies in the PNS, but the extent of PNS involvement in mice with GM1-gangliosidosis has not been investigated (Read et al., 1976; Yamano et al., 1983; Iwamasa et al., 1987; Shapiro et al., 2008; Jain et al., 2010; NINDS, 2011).

The goal of the current study was to determine if the content and composition of lipids and the structure of myelin were altered in the optic and sciatic nerves of *β-gal* −/− mice. We found that these nerves had less myelin, and an increase in GM1 ganglioside and GA1. The optic nerves of *β-gal* −/− mice had additional lipid and myelin structural abnormalities. These data suggest that deficiency of *β-gal* has a greater effect on the myelin of the optic nerves than of the sciatic nerves. The combination of lipid analysis and XRD has provided a better understanding of the neurochemical pathologies affecting the nerves of the CNS and PNS in GM1-gangliosidosis that may relate to the ocular phenotype (blindness, discoloration of the fovea, and optic neuropathy) of the disease.

## Materials and Methods

### Animals

B6/129 Sv mice, heterozygous for the β-galactosidase gene (*β-gal* +/−) were obtained from Saint Jude Children’s Research Hospital, Nashville, TN, USA (Dr. A. d’Azzo). These mice were generated by homologous recombination and embryonic stem cell technology, as previously described (Hahn et al., 1997). Sibling matings of the B6/129 Sv mice heterozygous for the *β-gal* knockout allele (+/−) were used to produce *β-gal* −/− mice. Male and female wild-type mice (*β-gal* +/+) and heterozygous mice (*β-gal* +/−), were used as controls (*β-gal* +/?). The mice were maintained through brother–sister inbreeding and kept in the Animal Care Facility of Boston College with all procedures in strict adherence with the NIH guide for the care and use of laboratory animals and approved by the Institutional Animal Care and Use Committee. The mice were housed in plastic cages with Sani-chip bedding (P.J. Murphy Forest Products Corp., Montville, NJ) and kept on a 12-hr light/dark cycle at approximately 22℃.

### Mouse Genotyping

DNA was isolated from ∼2 mm of mouse tail using the Wizard Genomic DNA purification Kit (Promega, Madison, WI) tail tissue protocol. Polymerase chain reaction amplification was performed using 1 µL of DNA (∼50–100 ng). The polymerase chain reaction amplification of the *β-gal* gene was set up as follows: 5 µL of 5× GoTaq Buffer, 0.3 µL dNTPs (10 mM mix), 10 μM *β-gal* gene forward primer (5'-ACACACAGGTTGAGAATGAGTACGG-3'), 10 μM *β-gal* reverse primer (5'-ACACACACCGACCTGTTCCAAAATC-3'), 10 μM neomycin-resistant (*Neo*) gene forward primer (5'-GTCACGACGAGATCCTCGCCGTC-3'), 10 μM *Neo* gene reverse primer (5'-GTCCGGTGCCCTGAATGAACTGC-3'), 0.25 µL GoTaq DNA Polymerase (Promega), and brought up to 25 µL with dH_2_O. The *β-gal* forward and reverse primers amplified a 200 bp fragment from the wild-type allele, whereas the *Neo* forward and reverse primer amplified a 500 bp fragment from the disrupted allele. The DNA was amplified using the following protocol: Initial denaturation 95℃ for 2 min, followed by 35 cycles of denaturation at 94℃ for 1 min; annealing 63℃ for 1 min; extension at 72℃ for 1 min; and a final extension at 72℃ for 10 min following the last cycle.

### Tissue Processing

All mice were sacrificed by cervical dislocation. For lipid isolation: Optic and sciatic nerves were isolated from each mouse and immediately frozen on dry ice, then stored at −80℃ until ready to use. Nerves were pooled from 11 to 20 mice (22 to 40 nerves) for each sample. Three sets of pooled samples were analyzed for each genotype [wild type (+/+), heterozygous (+/−), knockout (−/−)] and age (7 and 10 months). For XRD analysis: Optic and sciatic nerves were bathed with physiological saline (pH 7.4) during dissection, tied off with surgical silk, and immediately placed in fresh saline, as previously described (Avila et al., 2005; Agrawal et al., 2009). The nerves were inserted into 0.5 mm and 0.7 mm quartz capillaries (Charles Supper Co., Natick, MA), for optic and sciatic nerves, respectively, which were filled with saline and sealed at both ends with paraffin wax and enamel. XRD analysis was performed immediately after dissection, as described below.

### Lipid Isolation, Purification, and Quantitation

Complete lipid isolation, purification, and quantitation have been previously described (Seyfried et al., 1978; Hauser et al., 2004; Kasperzyk et al., 2004; Heinecke et al., 2011) and are briefly described as follows. Lipids were extracted from lyophilized nerve tissue. Neutral lipids and acidic lipids were separated using DEAE-Sephadex (A-25, Pharmacia Biotech, Upsala, Sweden) column chromatography as previously described (Macala et al., 1983; Heinecke et al., 2011). The entire neutral lipid fraction was collected and contained cerebrosides and asialo-ganglioside GM1 (GA1). Acidic lipids were eluted from the column and contained gangliosides and sulfatides. Neutral lipids were dried using the EZ-2 evaporator (Genevac, Gardiner, NY) and resuspended in chloroform (CHCl_3_): methanol (CH_3_OH) (1:1 by volume).

The acidic lipids, eluted from the DEAE-Sephadex, were dried by rotary evaporation and resuspended in CHCl_3_: CH_3_OH (1:1 by volume). CHCl_3_ and water were added to the sample to partition gangliosides in the upper phase and acidic phospholipids in the lower phase (Folch et al., 1957; Seyfried et al., 1978). The acidic phospholipid fraction was evaporated under a stream of nitrogen gas (N_2_) and resuspended in CHCl_3_: CH_3_OH (1:1 by volume). The amount of sialic acid in the ganglioside fraction was determined by a modified resorcinol assay before and after base treatment and desalting (Svennerholm, 1957). *N*-acetylneuraminic acid (Sigma, St. Louis, MO) was used as a standard curve for total ganglioside analysis. Samples and standards were analyzed in the Shimadzu UV-1601 ultraviolet-visible spectrophotometer (Shimadzu, Kyoto, Japan). The ganglioside fraction was further purified with base treatment and desalting after Folch partitioning (Hauser et al., 2004; Kasperzyk et al., 2004; Heinecke et al., 2011). Gangliosides were eluted from C18 reverse-phase Bond Elute columns (Varian, Harbor City, CA), evaporated under N_2_, and resuspended in CHCl_3_: CH_3_OH (1:1 by volume).

### High-Performance Thin-Layer Chromatography

High-performance thin-layer chromatography (HPTLC) was used to analyze neutral lipids, acidic phospholipids, and gangliosides according to previously described methods (Ando et al., 1978; Seyfried et al., 1978; Macala et al., 1983; Kasperzyk et al., 2004). Lipids were spotted on 10 × 20 cm, for gangliosides, or 20 × 20 cm, for neutral and acidic lipids, Silica gel 60 HPTLC plates (E. Merck, Darmstadt, Germany): 1.5 µg sialic acid for gangliosides, 80 µg nerve dry weight for neutral lipids, and 230 µg nerve dry weight for acidic lipids. Purified lipid standards (Matreya, Inc, Pleasant Gap, PA and Sigma, St. Louis, MO) were spotted on plates at 2, 4, and 8 µg, where the concentration is equivalent to the amount of each lipid per standard lane; except for the GA1 standard, which was spotted at 1, 2, and 4 µg.

For gangliosides, the HPTLC plates were developed with CHCl_3_: CH_3_OH: 0.02% calcium chloride (55:45:10 by volume), and the bands were visualized with resorcinol spray and burning at 100℃ for 10 min (Hauser et al., 2004; Kasperzyk et al., 2004). The total brain ganglioside distribution was normalized to 100%, and the percentage distribution values were used to calculate sialic acid concentration of individual gangliosides as previously described (Seyfried et al., 1982). For neutral and acidic phospholipids, the plates were developed with CHCl_3_: CH_3_OH: acetic acid: formic acid: water (35:15:6:2:1 by volume) to a height of either 10 cm or 12 cm, respectively, and then both were developed to the top with hexanes: diisopropyl ether: acetic acid (65:35:2 by volume) as previously described (Macala et al., 1983; Seyfried et al., 1984). The neutral and acidic lipids were visualized with 3% copper acetate: 8% phosphoric acid spray and heating at 160℃ for 7 min.

### Densitometry

Individual lipid bands were analyzed quantitatively by scanning the plates using a Camag TLC scanner 4 (Wilmington, NC), which is controlled by winCATS, Planer Chromatograpy Manager software (Muttenz, Switzerland). The HPTLC plates were placed face up on the scanner sample tray. Deuterium and tungsten-halogen lamps were used to visualize bands in the 190–450 nm range and the 350–900 nm range, respectively. Gangliosides were scanned at 580 nm wavelength, and neutral and acid lipids were scanned at 328 nm wavelength. Single-level calibration mode measured absorption for the evaluation of peak height and area. The total lipid distribution per lane of each plate was normalized to 100%, and the percentage distribution values were determined. The percent distribution of total gangliosides was used to calculate sialic acid concentration of individual gangliosides (Seyfried et al., 1982; Macala et al., 1983). Neutral and acidic lipids were calculated from the standard curve (Macala et al., 1983).

### X-Ray Diffraction

XRD experiments and analysis were conducted using our standard protocols (Avila et al., 2005; Agrawal et al., 2009), which are briefly described as follows. All diffraction experiments were carried out using nickel-filtered, single-mirror focused Cu Kα radiation from a fine-line source on a 3.0 kW Rigaku X-ray generator (Rigaku/MSC Inc., The Woodlands, TX) operated at 40 kV by 14 to 22 mA. The XRD patterns were recorded for 1 hr using a linear, position-sensitive detector (Molecular Metrology, Inc., Northampton, MA) and analyzed using PeakFit (Jandel Scientific, San Rafael, CA).

The positions of the intensity maxima (Bragg peaks) in the diffraction patterns were used to calculate the myelin period (*d*). Background intensity (*B*), approximated as a polynomial curve, was subtracted from the total intensity (*M* + *B*), and the total integral area of the Bragg peaks coming from the myelin (*M*) was obtained. The relative amount of myelin or [*M*/(*M* + *B*)] is calculated by dividing the total intensity coming form the multilamellar myelin (*M*, or the peak intensities above background) by the total intensity coming from the volume of nerve subtended by the X-ray beam (*M* + *B*; Avila et al., 2005).

### Statistical Analysis

All XRD values for the *β-gal* +/? and *β-gal* −/− mice were presented as mean ± *SD*, and all neurochemical values for the *β-gal* +/? and *β-gal* −/− mice were presented as mean ± *SE*. All data were analyzed for significance using the two-tailed Student’s *t* test. A value of *p* ≤ .05 was regarded as statistically significant.

## Results

The objective of this study was to determine if the content and composition of lipids and the structure of myelin were affected in the optic and sciatic nerves of the knockout mouse model of GM1-gangliosidosis. HPTLC and XRD were used to analyze the optic and sciatic nerves in the control and knockout mice.

### Lipid Analysis

#### Optic nerves

**Figure 1.**
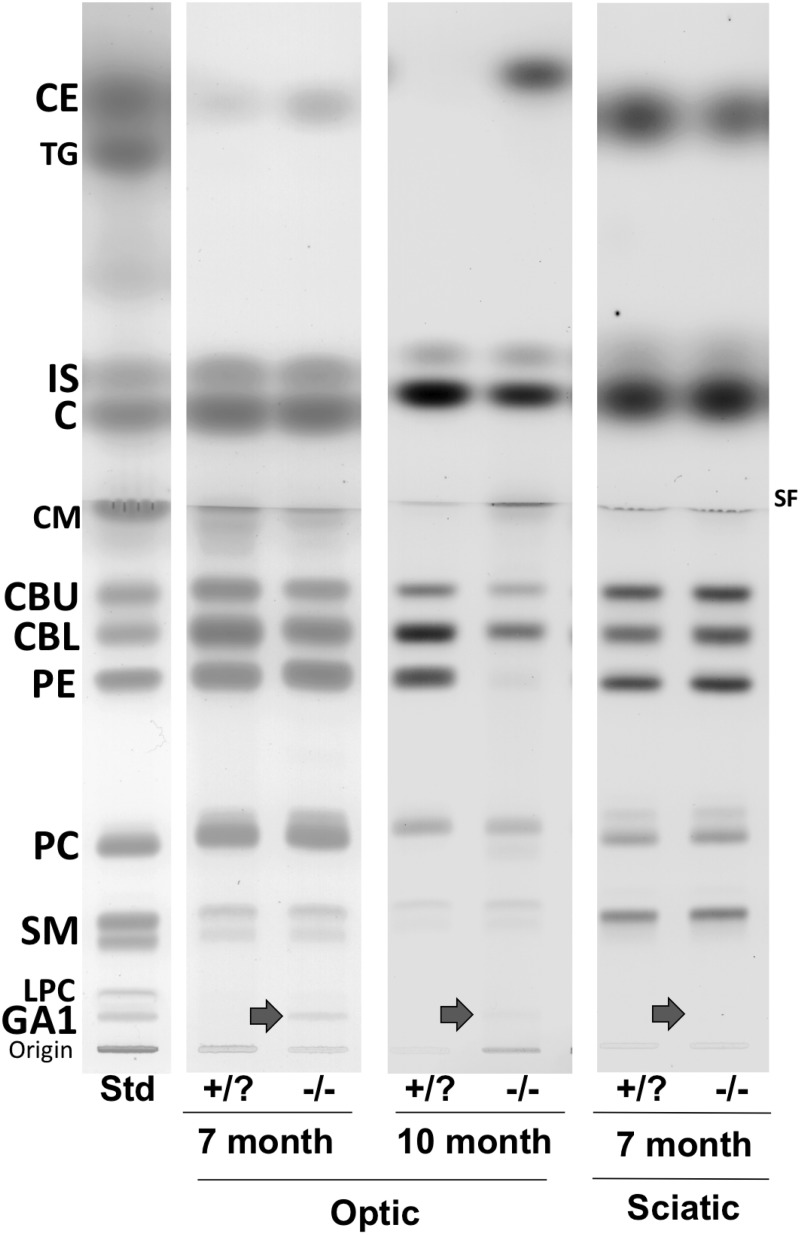
High-performance thin-layer chromatograms of neutral lipids in the optic and sciatic nerves of *β-gal* −/− and +/? mice. Representative samples for each age group and tissue type are presented. The amount of total lipids spotted per lane was equivalent to approximately 80 µg nerve dry weight. The plate was developed and the lipid bands visualized as described in Materials and Methods section. *Std* indicates 4 µg of neutral lipid standards and 2µg of GA1 standard. CE = cholesterol ester; TG = triacylglycerol; IS = internal standard (oleoyl alcohol); C = cholesterol; CM = ceramide; CBU = cerebroside upper band; CBL = cerebroside lower band; PE = phosphatidylethanolamine; PC = phosphatidylcholine; SM = sphingomyelin; LPC = lysophosphatidylcholine; SF = solvent front. The arrows indicate the presence of GA1 in the specific samples. Optic nerve contained no visible TG and sciatic nerves contained no visible CE.

**Figure 2.**
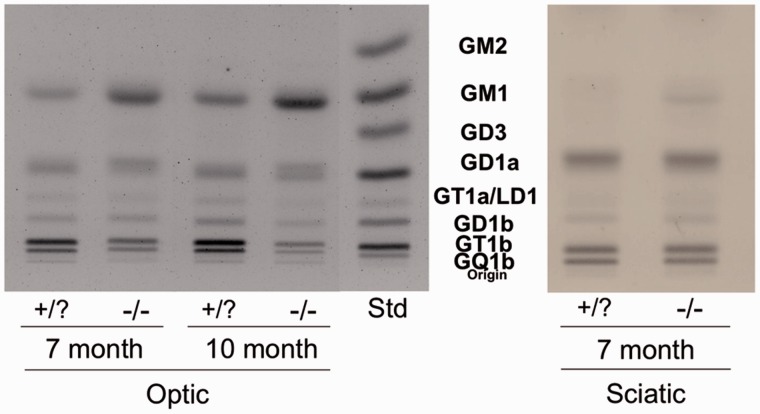
High-performance thin-layer chromatogram of gangliosides in the optic and sciatic nerves of *β-gal* −/− and +/? mice. Representative samples for each age group and tissue type are presented. Approximately 1.5 µg of ganglioside sialic acid were spotted per lane. *Std*, ganglioside standards for the labeled gangliosides; gangliosides GM2 and GD3 were not visualized in the nerve lipids. The plate was developed and the lipid bands visualized as described in Materials and Methods section.

The average weight per optic nerve was significantly lower in the *β-gal* −/− mice than in the *β-gal* +/? mice at 7 and 10 months (Table 1). The content levels of total gangliosides and GA1 were significantly greater in the optic nerves of the *β-gal* −/− mice than in the *β-gal* +/? mice (Table 1 and Figure 1). GM1 ganglioside increased in the optic nerves of 7- and 10-month old *β-gal* −/− mice by 50% compared with the *β-gal* +/? mice (Table 2 and Figure 2). There was a corresponding decrease of the more complex gangliosides GT1b and GQ1b in the optic nerves of *β-gal* −/− mice, compared with *β-gal* +/? mice. The gangliosides decreased by 47% (GT1b) and 41% (GQ1b) in 7-month old mice, and by 52% (GT1b) and 54% (GQ1b) in 10-month old mice. Ganglioside GD1a increased by 11% in 10-month old *β-gal* −/− compared with the *β-gal* +/? mice.

**Table 1.** Glycosphingolipid Content in Optic and Sciatic Nerves of *β-gal* Mice^a^.

Nerve type	Genotype	Age (months)	*N* ^b^	Average weight/nerve (µg)	µg SA/100 mg dry weight^c^	mg GA1/100 mg dry weight^d^
Optic	+/?	7	5	0.23 ± 0.00	126 ± 4	0.00 ± 0.00
	−/−		3	0.18 ± 0.00*	162 ± 3*	1.08 ± 0.08*
	+/?	10	3	0.28 ± 0.01	144 ± 3	0.00 ± 0.00
	−/−		3	0.15 ± 0.01*	197 ± 7*	1.22 ± 0.10*
Sciatic	+/?	7, 10	8	1.12 ± 0.04	39 ± 6	0.00 ± 0.00
	−/−		6	1.04 ± 0.04	44 ± 6	0.15 ± 0.04*

*Note.* Asterisks indicate that the value is significantly different from that of the control mice at * *p* < .01 as determined by the two-tailed *t* test.

a Values represent the mean ± *SE*.

b *N* = the number of independent samples analyzed, where 22 to 40 nerves were pooled for each sample.

c SA = sialic acid, values determined by resorcinol assay.

d Values determined from densitometric scanning of HPTLC plates as shown in Figure 1.

**Table 2.** Ganglioside Distribution in the Nerves of *β-gal* Mice^a^.

Ganglioside (total content)	Optic nerve				Sciatic nerve	
	7 month		10 month		7, 10 months	
	+/?	−/−	+/?	−/−	+/?	−/−
GM1	25.3 ± 0.8	51.5 ± 0.9*	25.6 ± 1.8	50.9 ± 1.9*	5.3 ± 0.3	14.6 ± 2.6*
GD1a	21.8 ± 0.5	19.4 ± 0.6	17.6 ± 0.2	19.8 ± 1.2*	34.8 ± 0.4	32.5 ± 0.8
GT1a/LD1	3.5 ± 0.2	3.6 ± 0.3	4.3 ± 0.7	2.8 ± 0.2	3.0 ± 0.2	3.0 ± 0.4
GD1b	7.0 ± 0.2	6.1 ± 0.4	10.1 ± 0.3	6.5 ± 0.3	5.2 ± 0.1	5.5 ± 0.4
GT1b	24.7 ± 1.3	13.0 ± 0.6*	27.6 ± 0.9	13.3 ± 1.3*	25.3 ± 0.7	21.3 ± 0.8
GQ1b	13.8 ± 0.3	8.2 ± 0.2*	16.6 ± 0.5	7.6 ± 0.6*	19.3 ± 0.5	16.1 ± 0.9

*Note.* Asterisks indicate that the value is significantly different from that of the control mice at * *p* < .01 and as determined by the two-tailed *t* test.

a Values determined from densitometric scanning of HPTLC plates, as shown in Figure 2, are expressed as percent distribution of ganglioside and represent the mean ± *SE*. The number of independent samples analyzed per nerve type and age group is listed in Table 1.

**Figure 3.**
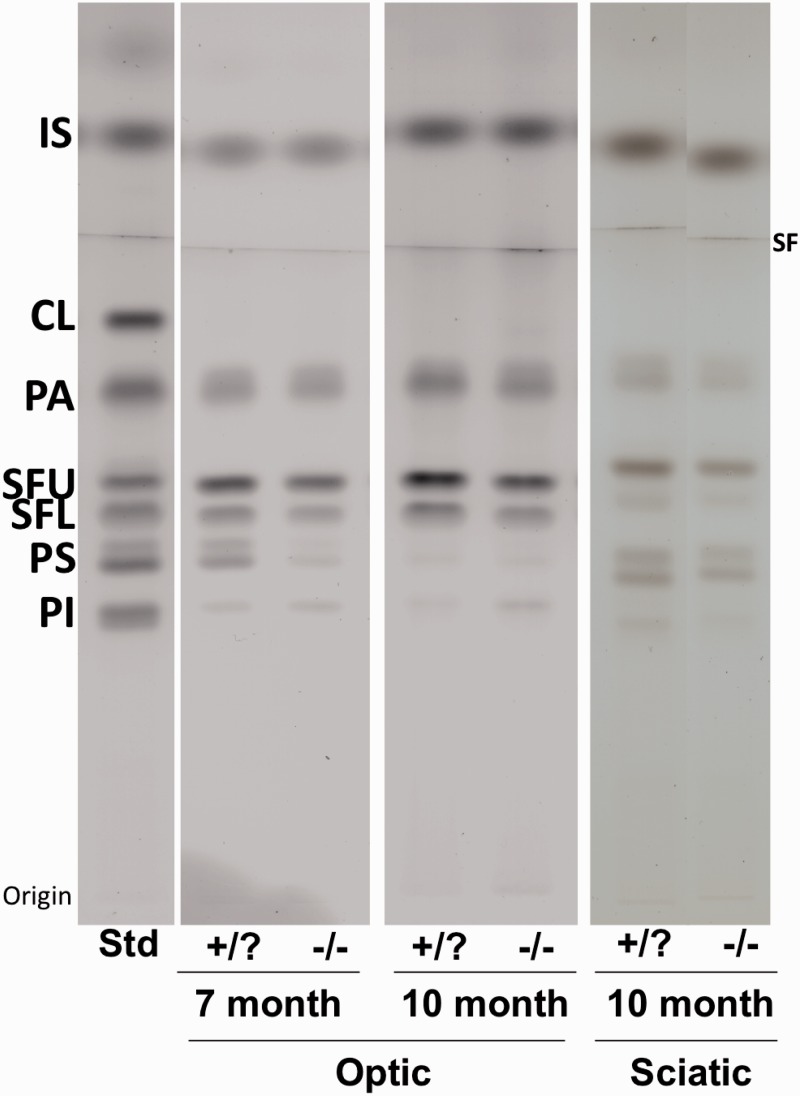
High-performance thin-layer chromatogram of acidic lipids in the optic and sciatic nerves of *β-gal* −/− and +/? mice. Representative samples for each age group and tissue type are presented. The amount of total lipids spotted per lane was equivalent to approximately 230 µg nerve dry weight for the acidic lipids. The plate was developed and the lipid bands visualized as described in Materials and Methods section. *Std*, 4 µg acidic lipid standards. IS = internal standard (oleoyl alcohol); CL = cardiolipin; PA = phosphatidic acid; SFU = sulfatide upper band; SFL = sulfatide lower band; PS = phosphatidylserine; PI = phosphatidylinositol; SF = solvent front.

The qualitative and quantitative distribution of neutral lipids (Figure 1) and acidic lipids (Figure 3) in the optic nerves from 7- and 10-month old mice are shown in Table 3. Total cerebrosides were decreased by 32% at 7 months and by 48% at 10 months in the optic nerves of *β-gal* −/− mice compared with *β-gal* +/? mice. Cholesteryl esters were increased significantly, whereas no substantial difference in cholesterol was detected in *β-gal* −/− mice at both ages. Phosphatidylethanolamines were reduced in *β-gal* −/− mice at 10 months. Sulfatides decreased by 24% at 7 months and 32% at 10 months in the optic nerves of *β-gal* −/− mice compared with *β-gal* +/? mice. Additional acidic lipids, phosphatidic acid, phosphatidylserine, and phosphatidylinositol increased with some variability between samples but showed no overall differences between the *β-gal* −/− and *β-gal* +/? mice (data not shown).

**Table 3.** Lipid Distribution in the Optic and Sciatic Nerves of *β-ga*l Mice^a^.

	Concentration (mg lipid/100 mg dry weight)^b^					
	Optic				Sciatic	
	7 month		10 month		7, 10 month	
Lipids	+/?	−/−	+/?	−/−	+/?	−/−
*Neutral*						
Cholesterol ester	1.3 ± 0.1	2.2 ± 0.1*	0.6 ± 0.1	6.5 ± 0.3**	ND	ND
Triacylglycerol	ND	ND	ND	ND	6.8 ± 0.7	6.8 ± 1.3
Cholesterol	10.0 ± 0.2	10.1 ± 0.4	11.1 ± 0.9	10.4 ± 0.9	7.0 ± 0.7	7.2 ± 1.0
Cerebrosides	12.0 ± 0.4	8.2 ± 0.4**	11.4 ± 0.1	5.9 ± 0.2**	6.4 ± 0.5	7.2 ± 1.0
Phosphatidylethanolamine	11.1 ± 0.7	10.4 ± 0.5	8.6 ± 0.4	1.2 ± 0.1**	5.3 ± 0.4	6.0 ± 0.5
Phosphatidylcholine	7.2 ± 0.3	7.1 ± 0.1	5.8 ± 0.4	5.5 ± 0.6	3.4 ± 0.2	3.9 ± 0.3
Sphingomyelin	1.6 ± 0.1	1.8 ± 0.1	1.2 ± 0.1	1.1 ± 1.3	2.9 ± 0.3	3.4 ± 0.6
*Acidics*						
Sulfatides	3.7 ± 0.1	2.8 ± 0.2*	3.8 ± 0.1	2.6 ± 0.1**	1.8 ± 0.1	1.6 ± 0.2

*Note.* Asterisks indicate that the value is significantly different from that of the control mice at *p < .02 and **p < .001, as determined by the two-tailed *t* test.

a Values determined from densitometric scanning of HPTLC plates, as shown in Figures 1 and 3, represent the mean ± *SE*. The number of independent samples analyzed per nerve type/age group is listed in Table 1.

b Values are expressed as mg of each lipid/100 mg dry weight of the total sample.

#### Sciatic nerve

There were no significant differences in the lipid content of the sciatic nerves between 7 and 10 months of age, so these two groups were combined. No differences were observed in the average weight per nerve, or the content of sialic acid, neutral, or acidic lipids (Tables 1 and 3, and Figures 1 and 3). GA1 was detected in the sciatic nerves of *β-gal* −/− mice (Figure 1 and Table 1). There was a 64% increase in GM1 ganglioside in the sciatic nerves from 7- and 10-month old *β-gal* −/− mice (Figure 2 and Table 2). No other differences in ganglioside content were found.

### XRD Analysis

**Figure 4.**
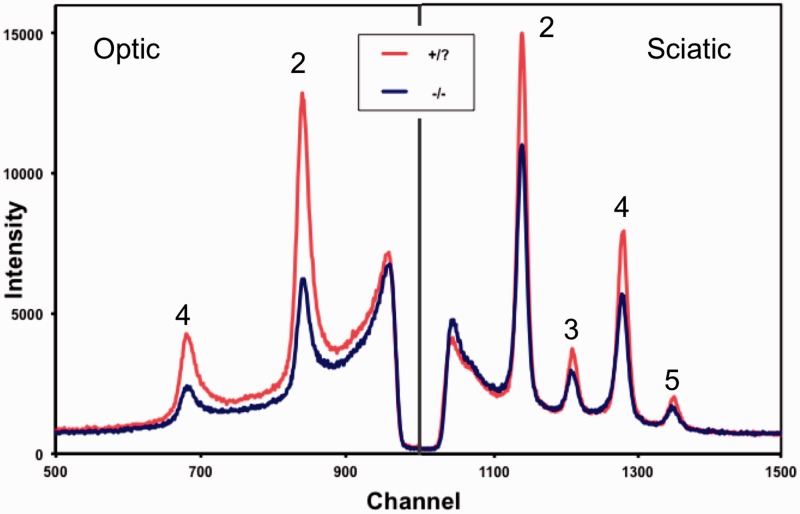
X-ray diffraction from optic and sciatic nerves from *β-gal* mice. Representative examples of data for optic (left) and sciatic (right) nerves from *β-gal* +/? and *β-gal* −/− are shown. Myelin scatter was significantly weaker in optic nerves (*p* < .001) and marginally weaker in sciatic nerves (*p* < .03) of *β-gal* −/− mice compared to nerves from *β-gal* +/? mice. The Bragg orders for the X-ray peaks are indicated by 2–5.

**Figure 5.**
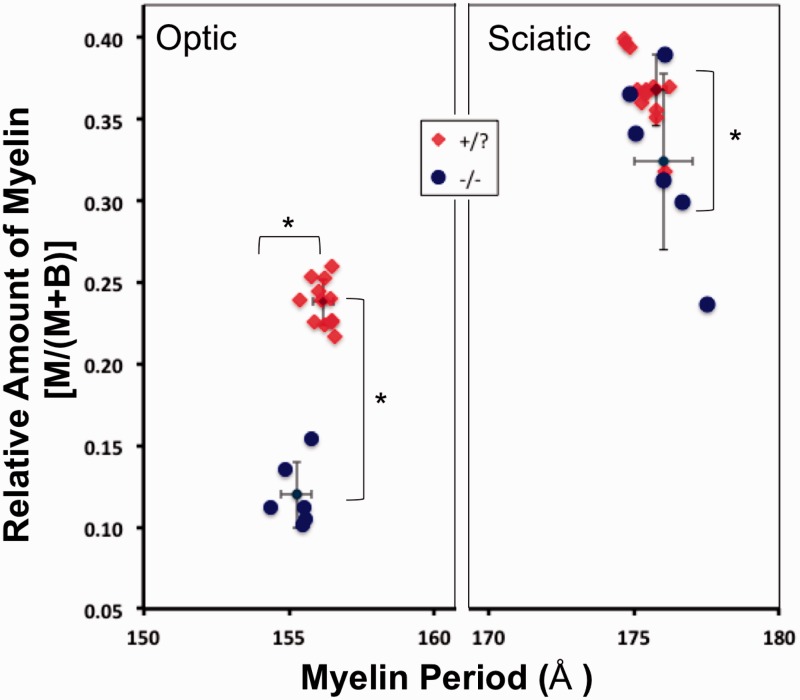
XRD analysis of myelin content and myelin periodicity in *β-gal* −/− and *β-gal* +/? mice. The fractional amount of scatter by compact myelin (M) compared with the relative amount of total X-ray scatter (*M + B*) is plotted against the myelin period, in Angstroms (Å). The mean value and standard deviations are indicated for each group of data (*N* = 12 nerves per group for *β-gal* +/?, and six nerves per group for *β-gal* −/−). The relative amount of myelin was significantly lower in the optic and sciatic nerves of *β-gal* −/− (○) mice compared to *β-gal* +/? (♦) mice. Myelin periodicity was significantly less in the optic nerves of *β-gal* −/− (○) mice than in *β-gal* +/? (♦) mice. The sciatic nerves of *β-gal* −/− (○) and *β-gal* +/? (♦) mice showed no significant differences in periodicity. Asterisks indicate statistical significance of *p* < .003, based on Student’s two-tailed unpaired *t* test.

Fresh optic and sciatic nerves were dissected from *β-gal* +/? and *β-gal* −/− mice and evaluated by XRD analysis. Based on the relative strengths of the diffraction patterns (Figure 4), the relative amount of myelin was about 50% lower in the optic nerve and 10% lower in the sciatic nerve of *β-gal* −/− mice compared with the *β-gal* +/? mice (Table 4 and Figure 5). Myelin periodicity in optic nerve was significantly lower in the *β-gal* −/− mice than in the *β-gal* +/? mice. No significant differences in the myelin period in sciatic nerves were detected.

**Table 4.** X-Ray Diffraction Analysis of *β-gal* Mice^a^.

	Optic nerve		Sciatic nerve	
	+/?	−/−	+/?	−/−
Age (days)	207 ± 15	210 ± 20	207 ± 15	210 ± 20
*M*/(*M* + *B*)^b^	0.25 ± 0.01	0.12 ± 0.02**	0.37 ± 0.02	0.32 ± 0.05*
*d* ^c^	156.2 ± 0.4	155.3 ± 0.5**	175.4 ± 0.5	176.0 ± 1.0

*Note*. Asterisks indicate statistical significance where *p < .03 and **p < .001, based on Student’s two-tailed *t* test.

a Values represent mean ± *SD*. *N* = 12 nerves per group for *β-gal* +/?, and 6 nerves per group for *β-gal* −/−. Optic and sciatic nerves were from the same mice.

b *M*/(*M* + *B*) = The myelin content of fresh nerves, based on the ratio of the X-ray diffraction scatter of the peaks over the total scatter, as shown in Figure 4.

c *d* = the periodicity of the peaks, as shown in Figure 2, and is displayed as angstroms (Å).

## Discussion

Our results show that loss of GM1 *β-gal* activity is associated with significant abnormalities in the content and composition of optic nerve and sciatic nerve lipids in the *β-gal* −/− mice. GM1 ganglioside, cerebrosides, and sulfatides have been used as markers for myelin content and composition (O’Brien and Sampson, 1965; Yu and Yen, 1975; Seyfried and Yu, 1980; Zalc et al., 1981; Coetzee et al., 1996; Muse et al., 2001). Previous analyses in the brains of humans and animal models with GM1-gangliosidosis have shown alterations in myelin-enriched lipids as well as in levels of cholesteryl esters and plasmalogen ethanolamines (Kasperzyk et al., 2004; Kasperzyk et al., 2005; Broekman et al., 2007; Baek et al., 2010). Electron microscopy and histopathology of GM1-gangliosidosis brains have shown reduction in the amount of nerves present in different brain regions (Tessitore et al., 2004; van der Voorn et al., 2005). Storage material in CNS and PNS nerves of GM1-gangliosidosis mice has been shown to initiate an endoplasmic reticulum (ER) stress response leading to apoptosis (Tessitore et al., 2004; Sano et al., 2009; Platt et al., 2012; Lupachyk et al., 2013). Increases in both cholesteryl esters and lysoplasmalogen ethanolamines (from the hydrolysis of plasmalogen ethanolamines) are also known to lead to ER stress and apoptosis (Farooqui and Horrocks, 2001; Farooqui et al., 2006). Inflammation also plays a role in disease progression in both GM1 and GM2-gangliosidosis mice (Jeyakumar et al., 2003; Chrast et al., 2011). The results presented here indicate that abnormalities in myelin lipids are also present in the optic and sciatic nerves of *β-gal* −/− mice with GM1-gangliosidosis, though the abnormalities were markedly greater in the optic nerve than in the sciatic nerve.

Ganglioside content in myelin has been shown to increase with age, and the same trend was observed in the optic nerves of *β-gal* +/? mice between 7 and 10 months of age (Suzuki et al., 1967; Yu and Yen, 1975). The increase in ganglioside content was even greater among *β-gal* −/− mice compared with controls, due to the increase in GM1 content, as previously seen in whole brains (Hauser et al., 2004; Kasperzyk et al., 2005; Broekman et al., 2007; Baek et al., 2010). Accumulation of GM1 ganglioside and GA1 was observed in the optic and sciatic nerves of *β-gal* −/− mice. It is known that GM1 accumulates naturally with age in myelin (Ando et al., 2003; Hauser et al., 2004). As our data showed, cerebroside content was greater in optic nerve than in sciatic nerve, the difference between optic nerve and sciatic nerves for storage pathology could be due in part to the difference in myelin content. The optic nerves of *β-gal* −/− mice had additional ganglioside abnormalities not observed previously in the whole brain; specifically, reduction of GT1b and GQ1b and an increase in GD1a. However, while not consistent in all brain regions (cortex, cerebellum, brainstem, and spinal cord) or statistically significant, Baek et al. (2010) did report a reduction in GT1b and GQ1b and an increase in GD1a in the *β-gal* −/− mice compared with controls. GT1b and GD1a are known to reside in the plasma membrane of axons and contribute to axonal-myelin stability (Vyas et al., 2002; Schnaar, 2004; Jackman et al., 2009). An increase in GD1a might occur to compensate for the decrease in GT1b, in an attempt to maintain axonal-myelin integrity. The *N*-metyl-D-aspartate (NMDA) receptor is the primary pathway for calcium influx into the myelin of optic nerve and is associated with neurotoxicity when activated (Sucher et al., 1991; Micu et al., 2006; Shin et al., 2014). NMDA receptor has also been associated with neurotoxicity in retinal ganglion cell cultures (Sucher et al., 1991). GQ1b has been shown to regulate expression of the NMDA receptor protein, and reduction in GQ1b may reduce calcium influx and thus reduce potential neuronal damage (Micu et al., 2006; Shin et al., 2014). Our findings indicate that the *β-gal* deficiency in the *β-gal* −/− mice alters ganglioside composition, which could contribute to abnormalities in optic nerve function.

The cerebroside and sulfatide content was reduced in the optic nerves of *β-gal* −/− mice, in agreement with the previously observed reduction in these lipids in the whole brain of *β-gal* −/− mice (Baek et al., 2010). In addition, there was reduction of phosphatidylethanolamine at 10 months, and an increase in cholesteryl ester at 7 and 10 months in the optic nerves of *β-gal* −/− mice, as observed in humans (Suzuki et al., 1968; Kasama and Taketomi, 1986). An increase in cholesteryl esters correlates to an increase in inflammation and myelin breakdown in the nervous tissue (Yu et al., 1982; Paintlia et al., 2003; Mutka et al., 2010; Platt et al., 2012). The majority of phosphatidylethanolamine in myelin is in the form of plasmalogen ethanolamines (Farooqui and Horrocks, 2001). All ethanolamine lipids are resolved together by TLC analysis, so it was deduced that the reduction in the phosphatidylethanolamine band was a result of a primary reduction in plasmalogen ethanolamines. Reduction in cerebroside, sulfatide, and plasmalogen ethanolamines is known to affect stability in the paranodal junction and the interaction of the paranodal myelin with the axon, which could alter conduction velocity (Coetzee et al., 1996; Farooqui and Horrocks, 2001; Ishibashi et al., 2002; Marcus et al., 2006; Jackman et al., 2009; Chrast et al., 2011; Hayashi et al., 2013; Viader et al., 2013) . These data suggest that the optic nerve lipids were altered in ways that reduce myelin integrity. A reduction in myelin stability and conduction velocity could explain one aspect of the neuronal and visual abnormalities observed in GM1-gangliosidosis mice (Murray et al., 1977; Bieber et al., 1986; Denny et al., 2007; Baek et al., 2010).

Yamano et al*.* (1983) previously showed that GM1 ganglioside accumulated in the PNS before the spinal cord and brain in a human fetus with GM1-gangliosidosis. The increase of GM1 ganglioside and GA1 in the sciatic nerves parallels the increase of both glycosphingolipids in the optic nerves. Our results in the sciatic nerves of *β-gal* −/− mice are therefore consistent with the previously observed accumulation of GM1 ganglioside and GA1 in the PNS of patients (Suzuki et al., 1968; Iwamasa et al., 1987; Folkerth et al., 2000; Nada et al., 2011; NINDS, 2011). However, the additional lipid abnormalities in the optic nerves, compared with whole brain, were not observed in the sciatic nerves of *β-gal* −/− mice. Moreover, our preliminary unpublished data also show that BMP (bis-monoacylglycero-phosphate), a secondary storage material in gangliosidoses, is elevated in optic nerve but is not elevated in sciatic nerve of *β-gal* −/− mice. These findings are consistent with the greater amount of GM1 storage in optic nerve than in sciatic nerve.

XRD has been a useful technique in assessing and comparing the overall structure of internodal nerve myelin—that is, the relative amount of myelin and the quality and extent of compaction (Kirschner and Sidman, 1976; Chia et al., 1984; Inouye et al., 1985; Mateu et al., 1991; Karthigasan et al., 1996; Avila et al., 2005; Yin et al., 2006; Agrawal et al., 2009; Avila et al., 2010; Kirschner et al., 2010). The relative amount of myelin and the myelin periodicity in control optic and sciatic nerves examined here are consistent with previously published data (Mateu et al., 1991; Agrawal et al., 2009; Avila et al., 2010). This is the first time XRD analysis has been performed on the nerves in GM1-gangliosidosis animals, and our results show that abnormalities in myelin content and periodicity in the *β-gal* −/− mice are greater in optic nerve than in sciatic nerve, which was consistent with the data from the lipid analyses. Although biochemical abnormalities in peripheral nerves might contribute in part to the behavioral abnormalities seen in patients with GM2 storage disease (Salman et al., 2001), our results suggest that such abnormalities are not likely to be a major issue in the *β-gal* −/− mice, which have minimal lipid and structural abnormalities in sciatic nerve.

Retinal and visual defects have been found in mice and other animals with GM1-gangliosidosis (Read et al., 1976; Murray et al., 1977; Sheahan et al., 1978; Suzuki et al., 2001; Denny et al., 2007; Baek et al., 2010; NINDS, 2011). These abnormalities are characterized by ganglioside accumulation in the retinal ganglion cells and altered myelination of the optic nerve (Sheahan et al., 1978; Kaye et al., 1992; Shen et al., 1998; Folkerth et al., 2000; Muller et al., 2001; Di Rocco et al., 2005; Gururaj et al., 2005; Brunetti-Pierri et al., 2008). GM1 ganglioside accumulates in retinal ganglion cells in humans and mice with GM1-gangliosidosis (Emery et al., 1971; Weiss et al., 1973; Cogan et al., 1984; Bieber et al., 1986; Denny et al., 2007). These abnormalities contribute to the blindness seen in GM1-gangliosidosis (Baker and Lindsey, 1974; Hahn et al., 1997; Matsuda et al., 1997a, 1997b; Suzuki et al., 2001; Tessitore et al., 2004; Baek et al., 2010). Using an adeno-associated virus (AAV) vector thalamic gene delivery to correct storage in GM1-gangliosidosis mice, Baek et al. (2010) observed a significant reduction of GM1 ganglioside and GA1 accumulation in most CNS structures and an increase in survival, but motor impairment and blindness were not completely corrected. Retina and optic nerve differ in their lipid content, and gangliosides do not move from the retina to the optic nerve (Holm, 1972; Holm and Mansson, 1974). These findings together with our data suggest that the lipid and myelin structural abnormalities seen in the retina and the optic nerve can contribute at least in part to the visual defects associated with GM1-gangliosidosis.

To our knowledge, this is the first study demonstrating a reduction in the quantity and quality of myelin in the optic and sciatic nerves of mice with GM1-gangliosidosis. The neurochemical pathology was altered in the optic and sciatic nerves of these mice. Nerve weight, total gangliosides, GM1, GA1, cerebrosides, and sulfatides were altered not just in the brains but also in the individual nerves of the CNS. Combined with alterations in GD1a, GT1b, GQ1b, cholesteryl ester, and plasmalogen ethanolamine (represented by phosphatidylethanolamine) content, these lipid differences resulted in a reduction in the amount of myelin and myelin periodicity in the optic nerves. While PNS involvement was not as severe as in the CNS, the sciatic nerves did accumulate GM1 ganglioside and GA1, and had a reduction in myelin content. The results show that the degree of myelin structural abnormalities observed in optic nerve and sciatic nerve was correlated with the degree of lipid abnormalities in these nerves. It is not clear however, whether the minor lipid defects observed in sciatic nerve would contribute to the behavioral abnormalities seen in the *β-gal* −/− mice. Recent studies indicate that AAV gene therapy can target sciatic nerve in mice with GM2 storage (Cachon-Gonzalez et al., 2012). Future studies on therapeutic regimens for GM1-gangliosidosis should include the analysis of the optic and sciatic nerves as part of a comprehensive assessment of the extent of reversal of the phenotype after treatment.
